# Editorial: Lifestyle and self-management of chronic pain across the lifespan

**DOI:** 10.3389/fnhum.2023.1249897

**Published:** 2023-11-13

**Authors:** Carolina Sitges, Sergi García-Retortillo, Marian Van der Meulen, Ana M. González-Roldán

**Affiliations:** ^1^Department of Psychology, Research Institute of Health Sciences (IUNICS), Health Research Institute of the Balearic Islands (IdISBa), University of the Balearic Islands, Palma, Spain; ^2^Department of Health and Exercise Science (HES), Wake Forest University, Winston-Salem, NC, United States; ^3^Department of Behavioural and Cognitive Sciences, University of Luxembourg, Esch-sur-Alzette, Luxembourg

**Keywords:** fibromyalgia, quality of life, pain expectancy, event-related potentials, kinesiophobia, scoping review, back awareness, chronic low back pain

## 1 Introduction

Chronic pain is the leading cause of disability in industrialized countries and its prevalence increases throughout adulthood. The aim of this Research Topic is to explore the different factors influencing its development and maintenance across the lifespan. The articles included in this collection range from basic to clinical research, including practical applications to improve current assessment instruments and self-management practices. This collection of articles is comprised of three types of manuscripts (two research articles, one scoping review and one methodological paper) focusing on the following subtopics of chronic pain: (1) factors affecting quality of life in fibromyalgia (2) alteration in electrophysiological indices of pain expectation in fibromyalgia, (3) assessment and treatment of kinesiophobia in musculoskeletal pain conditions, and (4) proprioceptive acuity is core for back awareness in chronic low back pain.

## 2 Factors affecting quality of life in fibromyalgia

Fibromyalgia (FM) is characterized by the presence of chronic and widespread musculoskeletal pain, and other symptoms (such as fatigue, sleep disturbances, cognitive alterations, and mood disorders), which cause a high negative impact on quality of life (QoL). This Research Topic includes a study by Fernandez-Feijoo et al. that performed both linear and logistic regression analysis of these symptoms and other less explored factors that may also predict QoL, such as lifestyle (including diet, exercise, and tobacco use) and multi-medication (i.e., a medication pattern consisting of more than three different types of drugs classified as analgesics, anxiolytics, antidepressants, antipsychotics, sedatives, or others). They also compared subgroups created based on lifestyle and medication patterns. The results of this study have relevant clinical implications for improving QoL of patients with FM. The symptoms which most significantly predicted QoL (explaining 49% of the variance) were depression and anxiety. Medication and smoking also predicted lower QoL (explaining 14% of the variance). Moreover, patients who exercised regularly had improved QoL compared to patients who did not (regardless of the severity of FM). Overall, these results highlight the importance of increasing self-management practices focused on improving mood, but also sleep quality, reducing medication and tobacco use, and promoting a balanced diet and regular exercise.

## 3 Alteration in electrophysiological indices of pain expectation in fibromyalgia

Among cognitive alterations in FM, attentional biases or hypervigilance to pain leads patients to anticipate or interpret pain as a threat, promoting inadequate coping strategies (such as escape and avoidance behaviors). Therefore, these cognitive alterations seem to contribute to the origin, exacerbation and maintenance of increased pain perception and may even be involved in the severity of FM symptoms. From a basic research perspective, Barjola et al. recorded event-related potentials (ERPs) during an S1-S2 (or cue-target) paradigm, consisting of the presentation of an image (triangle or square) that predicted the occurrence of a (painful or non-painful) laser stimulation,_to explore the temporal dynamics of anticipatory attention to pain processing (or pain expectancy). Although no statistically significant differences were found in behavioral data (i.e., subjective pain ratings and reaction times), an abnormal pattern of pain expectancy (i.e., a decreased amplitude of an ERP component related to the anticipation of pain (posterior lCNV), and an enhanced amplitude of the P2 component related to increased stimulation intensity but not related to pain predictive cues) were observed in FM participants compared to healthy controls. The results of this study also have practical implications, as a better understanding of neural correlates of pain processing and modulation allows for the development of more effective coping strategies to improve QoL of chronic pain patients.

## 4 Assessment and treatment of kinesiophobia in musculoskeletal pain conditions

The fear-avoidance model of pain describes how negative and catastrophizing beliefs about pain could lead to a vicious cycle of fear, activity avoidance, and resultant disuse and distress. In line with this, when a painful event is perceived as threatening, it can lead to catastrophizing thoughts (e.g., the belief that movement and physical activity will result in further pain and injury). This fear of movement, or kinesiophobia, is common in patients with chronic pain, and is associated with increased pain intensity and disability. The knowledge gained from the scoping review by Bordeleau et al. about the assessment and treatment of kinesiophobia is highly relevant to evidence-based clinical practice. According to their results, the Tampa Scale of Kinesiophobia (TSK) is the most used tool for measuring kinesiophobia, but the Fear-Avoidance Components Scale (FACS), which is only starting to be used, seems to be the most suitable tool to date to assess the multiple components of fear of movement. The authors also point out that physical exercise is a key component of non-pharmacological interventions for musculoskeletal pain and that high levels of kinesiophobia may compromise its adherence.

## 5 Proprioceptive acuity is core for back awareness in chronic low back pain

Patients with chronic low back pain (CLBP) can present with different sensorimotor abnormalities, including body image disturbances (e.g., perceiving the back as fragile and vulnerable or feelings of exclusion, alienation, and rejection toward the back). Consequently, an increasing number of studies focuses on altered back awareness as a potential contributor to CLBP and a target for treatment. The Fremantle Back Awareness Questionnaire (FREBAQ) is a validated and reliable tool to assess back-specific altered body perception in patients with CLBP. The study developed by García-Dopico et al. presents a further analysis of the face/content validity of the Spanish version of the FREBAQ (FreBAQ-S), based on questions regarding the completeness, comprehensibility, time-to-complete adequacy, and time spent completing this questionnaire, in a large sample of CLBP patients and healthy controls. The authors also explored additional variables involved in back awareness suggested by the participants, categorized into four classes: neglect-like symptoms (e.g., alienation), proprioceptive acuity (e.g., posture, weight, or movement patterns), trunk shape and size, and psychological variables (e.g., attention to pain, fear). Their results showed that CLBP participants spent significantly more time on completing the questionnaire than controls, but no differences were found between groups regarding the time-to-complete adequacy. Regarding the back awareness-related variables, CLBP patients made more suggestions than healthy controls (77 vs. 7, respectively), most of them related to proprioceptive acuity.

In conclusion, this Research Topic aims to enhance our understanding of the multifaceted nature of chronic pain and its impact on individuals across the lifespan. The diverse range of topics covered provides valuable new insights for researchers, clinicians, and individuals living with chronic pain, offering opportunities to develop more effective interventions and improve quality of life for those affected by chronic pain conditions.

## Author contributions

CS redacted the first draft of the manuscript. All authors reviewed and improved the manuscript. All authors contributed to the article and approved the submitted version.

